# Dose–Response Effects of High-Intensity Interval Training on Body Fat, Blood Pressure, and Cardiorespiratory Fitness in Adolescents: A School-Based Randomized Controlled Trial with Responder Analysis

**DOI:** 10.3390/jfmk10040439

**Published:** 2025-11-13

**Authors:** Jarosław Domaradzki, Eugenia Murawska-Ciałowicz, Marek Popowczak, Katarzyna Kochan-Jacheć, Paweł Szkudlarek, Dawid Koźlenia

**Affiliations:** Faculty of Physical Education and Sport, Wroclaw University of Health and Sport Sciences, 51-612 Wroclaw, Poland; jaroslaw.domaradzki@awf.wroc.pl (J.D.); eugenia.murawska-cialowicz@awf.wroc.pl (E.M.-C.); marek.popowczak@awf.wroc.pl (M.P.); katarzyna.kochan-jachec@awf.wroc.pl (K.K.-J.); pawel.szkudlarek@op.pl (P.S.)

**Keywords:** high-intensity interval training, dose–response, responders, adolescents, Bayesian classification

## Abstract

**Background**: High-intensity interval training (HIIT) is effective for improving body composition and cardiorespiratory fitness, but individual variability in responsiveness remains a challenge. This study examined the dose–response effects of three HIIT session durations (6, 8, and 10 min) and whether previously non-responsive adolescents could benefit from a modified program. **Methods**: A total of 137 adolescents completed one of three school-based HIIT interventions. Body fat percentage (BF%), systolic and diastolic blood pressure, and maximal oxygen uptake (VO_2_max) were assessed before and after the intervention. Responders and non-responders were classified using a Bayesian approach. Statistical analyses included ANOVA, McNemar’s test, logistic regression, and generalized estimating equations. **Results**: All protocols improved outcomes, with the 10 min sessions producing the most consistent VO_2_max gains. No significant differences were observed for BF% or blood pressure. Individual analysis showed that more than half of the participants responded positively to training, depending on the outcome. Among prior non-responders, 70–100% showed improvements after the modified intervention. The number of previously non-responsive outcomes strongly predicted improvement (Odds Ratio > 2.4, *p* < 0.01). **Conclusions**: School-based HIIT can induce meaningful adaptations even in previously non-responsive adolescents. Individualized monitoring and adjustment of training dose may optimize responsiveness and support health promotion in youth.

## 1. Introduction

Our previous work [1] showed that two forms of high-intensity exercise—traditional high-intensity interval training (HIIT) and a plyometric-based variant (HIPT)—can lead to reductions in fat mass, a lower resting blood pressure, and improved cardiorespiratory fitness. However, the outcomes of HIIT interventions often differ depending on sex, baseline body mass index (BMI), and other intra-individual factors [2,3].

Considerable variability has been observed even when all participants follow the same training protocol [4,5,6]. This phenomenon—referred to as individual variability in response to exercise training (IVRET)—illustrates that, while group averages often indicate improvement, some individuals show minimal or no measurable adaptations [7]. Recognizing this variability is crucial, as overlooking non-responders may lead to misleading conclusions about a training program’s overall effectiveness.

Despite the overall benefits of HIIT, a key challenge remains: some individuals fail to exhibit adaptations due to an insufficient or suboptimal training stimulus. Inadequate dose or intensity may not reach the physiological threshold necessary for improvement, particularly in adolescents who display high inter-individual variability in trainability [8,9,10]. Moreover, few studies have examined the dose–response relationship in youth populations. Most have focused on group averages rather than individual trajectories [11], which risks overlooking non-responders—participants who fail to improve despite completing the program [10]. This limitation may mask important insights about program efficacy.

To address this gap, analyses should focus on identifying which training doses are sufficient to elicit responses in non-responding individuals and on refining intervention designs to optimize individual adaptations. Although numerous studies have demonstrated the beneficial effects of high-intensity interval training (HIIT) on cardiorespiratory fitness and cardiovascular health in youth, the optimal duration and structure of school-based HIIT sessions remain unclear. Most previous investigations implemented relatively long exercise bouts or controlled laboratory protocols, which differ from the practical realities of school environments. Therefore, in the present study, we evaluated a modified intervention differing in session duration (6, 8, and 10 min). This follow-up study is, to our knowledge, the first to re-examine previously non-responsive adolescents using a Bayesian responder framework to test whether individualized dose adjustments can overcome non-response. The aim was to examine effects on body fat percentage (BF%), systolic (SBP) and diastolic blood pressure (DBP), and cardiorespiratory fitness (CRF) in participants who had shown no improvements in these outcomes during a previous intervention conducted six months earlier. Specifically, we aimed to (1) compare the effects of the three experimental groups (6, 8, and 10 min sessions) with each other and with control groups; (2) identify responders (Rs) and non-responders (NRs) within each intervention using a Bayesian approach that accounts for individual variability and measurement error; (3) examine whether session duration is associated with the prevalence of Rs and NRs across outcomes, indicating a dose–response relationship; and (4) assess whether participants previously non-responsive in one or more outcomes improved after the modified intervention.

We hypothesized that (H_1_) a dose–response relationship exists between HIIT session duration and changes in BF%, SBP, DBP, and maximal oxygen uptake (VO_2_max) among prior non-responders; (H_1_a) effects differ significantly between durations; (H_1_b) each intervention outperforms its control; (H_1_c) the proportion of responders depends on session length; (H_1_d) previously non-responsive individuals improve in at least one outcome; and (H_1_e) session duration predicts the likelihood of becoming a responder. Hypotheses were structured hierarchically, with H_1_ representing the main assumption of a dose–response relationship and H_1_a–H_1_e addressing its specific, testable components.

## 2. Materials and Methods

### 2.1. Study Design

The initial trial lasted 8 weeks and included four groups: two experimental arms (HIIT and HIPT) and two corresponding control groups. In this school-based intervention [1], adolescents participated in two 45 min physical education sessions per week. The HIIT group performed bodyweight resistance exercises (e.g., squats, lunges, push-ups), while the HIPT group engaged in plyometric movements emphasizing explosive jumps and rapid repetitions. Each session followed a 20 s:10 s work-to-rest ratio, progressing from four to eight rounds across the 8 weeks, with effort intensity maintained at approximately 75–85% of maximal heart rate (HRmax). A six-month interval separated the initial and current trials, corresponding to one academic semester. During this period, participants attended only regular physical education classes without structured high-intensity training. This recovery period ensured comparable school and environmental conditions during reassessment. In the present follow-up intervention, the training structure and intensity were kept identical, while session duration was modified (6, 8, or 10 min) to examine the dose–response effect on previously non-responsive participants. The outcomes of this preliminary phase were used to determine the allocation of participants for the subsequent experimental study. A detailed description of recruitment, study procedures, and intervention protocols has been published elsewhere [1]. For the present analysis, only data from participants assigned to the experimental groups are included, as the primary aim was to quantify the delivered training load (TRIMP) and examine its association with health outcomes.

### 2.2. Clinical Trial Registration

This study was part of the national project Science for Society II, funded by the Polish Ministry of Science and Higher Education (project no. NdS-II/SP/0521/2023/01). It was prospectively registered at ClinicalTrials.gov (identifier: NCT06431230, 10 May 2024) under the acronym PEER-HEART (Physical Education dosE Response Health markErs Adolescents inteRval Training).

### 2.3. Ethics Committee

Ethical approval was granted by the Ethics Committee of the Wroclaw University of Health and Sport Sciences (decision no. ECUPE 33/2018, issued on 31 October 2018). All procedures followed the Declaration of Helsinki. Written informed consent was obtained from all participants and their legal guardians before enrollment.

### 2.4. Participants

Adolescents from the PEER-HEART project were included in the present analysis, specifically those assigned to the experimental arms (n = 145). Participants’ characteristics, including age, sex distribution, and group assignment, are summarized in Table 1. A detailed description of sample characteristics has been published previously [1]. Following the initial intervention, participants were classified as Rs if they showed improvements in BF%, SBP or DBP, or VO_2_max. Those without positive changes were classified as NRs.

Non-responders were randomly assigned (based on baseline ID codes) to four subgroups: three experimental groups—E6m (6 min HIIT), E8m (8 min HIIT), and E10m (10 min HIIT)—and one non-intervention control group (CON). An additional control group (CON-Rs) included individuals who had previously responded positively in all outcome variables. This group served as a stable reference to control for potential environmental or school-related effects and to verify whether improvements in previously non-responsive participants could be attributed to the modified intervention. The intervention design followed a dose–response approach based on session duration. Group sizes were as follows: E6m = 28 (M = 16, F = 12), E8m = 28 (M = 12, F = 16), E10m = 23 (M = 11, F = 12), CON = 26 (M = 11, F = 15), and CON-Rs = 32 (M = 16, F = 16).

**Figure 1:**
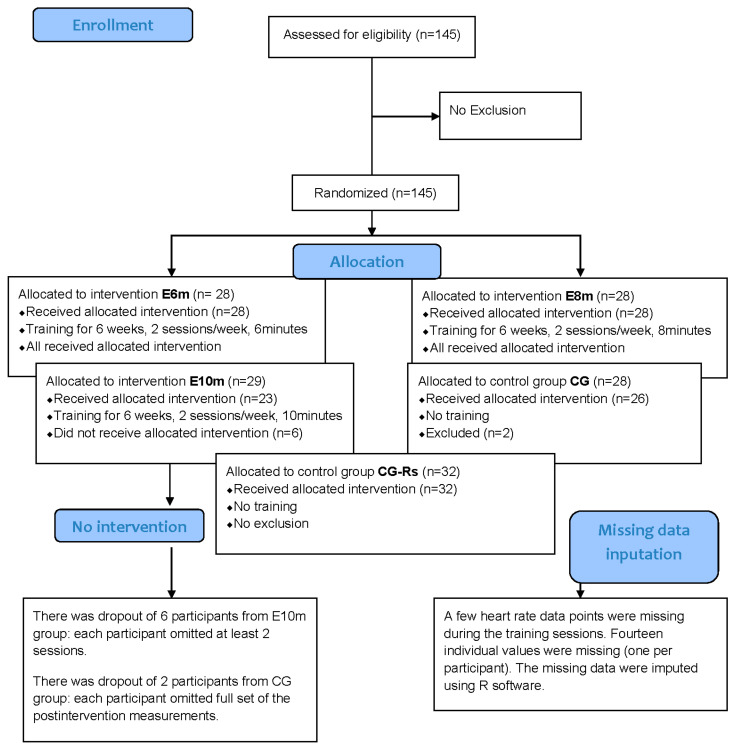
Flow diagram of the progress through all phases of data collection (CONSORT 2010).

Eight participants were excluded due to non-compliance: six from E10m (missed ≥2 sessions) and two from CON (missed all post-intervention assessments). In addition, 14 missing heart rate values recorded during HIIT sessions were statistically imputed. The allocation process is illustrated in Figure 1.

### 2.5. Intervention

The high-intensity interval HIIT intervention followed a Tabata protocol consisting of 20 s of maximal effort and 10 s of passive rest [12]. Sessions were delivered twice weekly during physical education classes (first and last lesson of each week). Participants were instructed to perform as many repetitions as possible during each work interval. If they could not sustain maximal effort, they were encouraged to continue at a self-selected pace until the set was completed.

Each session began with a standardized 10 min warm-up. The core program included four exercise pairs targeting major muscle groups and movement patterns:

Pair A: Squats and burpees with jump
Pair B: Alternating lunges and plank shoulder taps (with knee support if needed)
Pair C: Lateral squats and high knees
Pair D: Squat jumps and mountain climbers

The intervention was performed in AMRAP manner -as many repetitions as possible. The intervention lasted six weeks with two sessions per week. Three session durations were applied—6, 8, and 10 min—corresponding to the experimental groups. All sessions were conducted by qualified PE teachers who encouraged maximal effort during each 20 s work bout.

Exercise intensity was monitored using Polar heart rate monitors (Polar Electro, Kempele, Finland). For each participant, minimum, mean, and maximum heart rate values were recorded, and grand means were calculated to represent overall exercise intensity across the intervention.

### 2.6. Measurements

Detailed procedures have been reported previously [1]. In brief, assessments were conducted at two time points: before the 6-week intervention (pre-intervention) and immediately after its completion (post-intervention). All tests were performed indoors, under standardized conditions, between 8:00 a.m. and 1:00 p.m., on a single day for each group.

Anthropometry

Body height was measured to the nearest 0.1 cm using a GPM anthropometer (DKSH Ltd., Zurich, Switzerland) with participants standing barefoot. Body weight and BF% were assessed with the Tanita Inner Scan V analyzer (BC-601, Tanita Co., Tokyo, Japan). Before testing, participants were instructed to void their bladder, avoid excessive fluid intake, and follow their typical breakfast routine. All measurements were taken barefoot and shirtless, in standard anatomical position, at least 3 h after the last meal. Body mass index (BMI) was calculated as weight (kg) divided by height squared (m^2^).

Blood pressure

SBP and DBP were measured using an Omron BP710 automatic monitor (Omron Healthcare, Inc., Hoffman Estates, IL, USA), following the procedure described by Nasir et al. [13]. Cuff size was adjusted to arm circumference. After a 10 min seated rest, three consecutive measurements of systolic and diastolic blood pressure and heart rate were taken at 10 min intervals to ensure physiological stabilization. The mean of these readings was recorded as resting blood pressure and resting heart rate (HRrest), confirming that participants were in a stable baseline physiological state before testing. Assessments were performed at baseline, post-intervention, and follow-up.

Cardiorespiratory fitness

Cardiorespiratory fitness was assessed with the Multistage Fitness Test (MSFT). Heart rate was continuously monitored using Polar Verity Sense sensors (Polar Electro, Kempele, Finland) [14]. The MSFT consists of 20 m shuttle runs paced by beeps, starting at 8.5 km/h and increasing by 0.5 km/h each minute. Participants run back and forth until they can no longer maintain the pace. Maximal oxygen uptake (VO_2_max) was estimated with Ramsbottom’s equation [15]:

VO_2max_ = 3.46 × (L + SN/(L × 0.4325 + 7.0048)) + 12.2,

where L is the level, and SN is the number of shuttles.

### 2.7. Statistics

There were no missing data for anthropometry, body composition, blood pressure, or cardiorespiratory fitness. Missing heart rate values during intervention sessions (n = 14) were assumed to be missing completely at random (MCAR) [16,17] and imputed using the mice package in RStudio v.2025.05.1+513. Analyses were performed using Statistica (version 14.3.0) (frequentist tests), R (version 4.5.1) (multiple imputation and data preprocessing), and Python (version 3.13) (Bayesian responder classification), as each platform was suited to a specific analytic component.

Normality was tested with the Shapiro–Wilk test. Because BF% showed slight deviations, both parametric and non-parametric methods (Kruskal–Wallis and Wilcoxon signed-rank tests) were applied. When conclusions were consistent, ANOVA was used. Data are reported as means with 95% confidence intervals and standard deviations. Baseline homogeneity between groups was confirmed using two-way ANOVA (sex × group) for BF%, SBP, DBP, and VO_2_max, with no significant differences observed (all *p* > 0.05)

A two-way ANOVA assessed sex (male/female) and group (experimental/control) differences at baseline and in changes (∆) in outcomes: BF%, SBP, DBP, and VO_2_max. Intervention effects were analyzed with mixed-effects ANOVA (three-way: sex × group × time [pre, post]). Homogeneity and sphericity were checked with Levene’s and Mauchly’s tests; Greenhouse–Geisser corrections were applied when necessary. Effect sizes were reported as generalized partial eta squared (η^2^_p_^G^) and converted to Cohen’s d. Bonferroni correction was used for post hoc comparisons. For figures, we present change scores (Δ = POST − PRE) centered at zero; absolute PRE and POST values are reported in tables.

To examine individual variability, a Bayesian approach classified participants as responders (Rs), non-responders (NRs), or uncertain cases. This procedure followed the framework proposed by Mattioni Maturana et al. (2021) and enables a probabilistic, individualized interpretation of training responsiveness [18]. For each participant, a normal distribution was modeled around the change score, incorporating a 5.6% baseline-derived measurement error [18]. An 89% Highest Density Interval (HDI) was calculated and compared with the Region of Practical Equivalence (ROPE = ±0.05):

Responder (R): HDI below ROPE
Non-responder (NR): HDI above ROPE
Uncertain: HDI overlapping ROPE

Uncertain cases were further divided by ∆ direction: negative = R-like, zero/positive = NR-like. This classification was used in subsequent group comparisons and regression models.

To test whether previously non-responsive individuals improved after the modified intervention (Aim 4), three statistical strategies were applied:

• McNemar test—to compare binary response status before and after intervention.
• Logistic regression—to identify predictors of improvement (at least one positive response). Predictors: sex, intervention duration, prior non-responsiveness.
• Generalized Estimating Equations (GEEs)—to model binary response probability across repeated outcomes while accounting for intra-subject correlations [19]. Predictors included session duration (6, 8, 10 min), number of prior non-responsive outcomes (1–4), and their interaction. An exchangeable correlation structure and robust standard errors were applied.

Differences were considered statistically significant at *p* < 0.05 (equivalent to *p* ≤ α). Analyses were performed using Statistica 13.0, RStudio, and Python (Google Colab) for Bayesian responder classification.

## 3. Results

### 3.1. Descriptive Statistics

Table 1 shows baseline anthropometric characteristics by sex and group. Two-way ANOVA confirmed significant sex differences in body height (F = 69.65, *p* < 0.002) and body weight (F = 43.87, *p* < 0.001), but not in BMI (F = 0.12, *p* = 0.73). No main effects of the experimental factor or sex × group interactions were observed (*p* > 0.05). Although mean baseline VO_2_max values appeared slightly lower in the 8 and 10 min groups, these differences were not statistically significant (*p* > 0.05).

Table 2 presents baseline values for BF%, systolic and diastolic blood pressure, and VO_2_max. Mixed-effects ANOVA was used to compare outcomes across the three experimental and two control groups. Post hoc analyses with Bonferroni correction were applied, and results are reported for each outcome.

### 3.2. Body Fat Percentage

**Figure 2:**
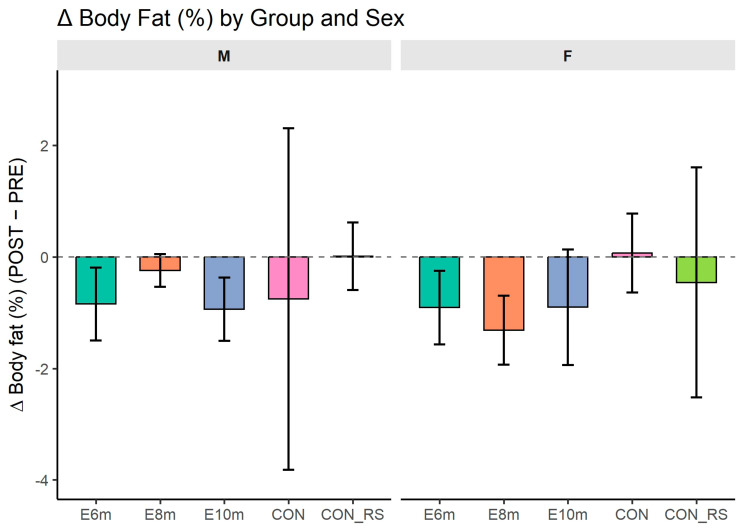
Changes (Δ = POST − PRE) in body fat (%) across intervention groups and sexes. Bars represent mean Δ values ± 95% CI; dashed line indicates zero change. Abbreviations: M—males; F—females; 6 min, 8 min, 10 min—experimental groups differing in session duration; CON—control group of non-responders; CON_Rs—control group of previous responders.

Figure 2 shows changes (Δ) between post- and pre-intervention BF% in experimental and control groups. ANOVA confirmed significant sex differences, with higher values in females than males (F(1,127) = 100.218, *p* < 0.001, η^2^_p_^G^ = 0.432, d ≈ 1.80). A main effect of time was also observed (F(1,127) = 11.730, *p* < 0.001, η^2^_p_^G^ = 0.003, d ≈ 0.11). However, no significant effects were found for HIIT duration (F(4,127) = 1.161, *p* = 0.331, η^2^_p_^G^ = 0.034, d ≈ 0.38), variant × time (F(4,127) = 0.645, *p* = 0.632, η^2^_p_^G^ = 0.001, d ≈ 0.05), sex × time (F(1,127) = 0.164, *p* = 0.686, η^2^_p_^G^ < 0.001, d ≈ 0.01), or the three-way interaction (F(4,127) = 0.720, *p* = 0.580, η^2^_p_^G^ = 0.001, d ≈ 0.06).

Post hoc comparisons (Bonferroni corrected) showed no significant pre–post changes in any group (all *p* = 1.000). Delta analyses confirmed no differences between groups (F = 0.64, *p* = 0.632) or between sexes (F = 0.16, *p* = 0.686), and no interaction effect (F = 0.72, *p* = 0.579).

### 3.3. Systolic Blood Pressure

**Figure 3:**
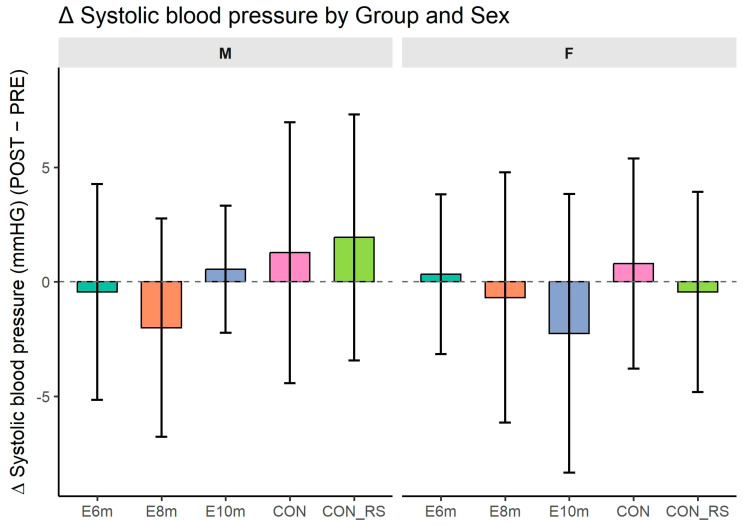
Changes (Δ = POST − PRE) in systolic blood pressure (sbp) across intervention groups and sexes. Bars represent mean Δ values ± 95% CI; dashed line indicates zero change. Abbreviations: M—males; F—females; 6 min, 8 min, 10 min—experimental groups differing in session duration; CON—control group of non-responders; CON_Rs—control group of previous responders.

Figure 3 shows changes (Δ) between post- and pre-intervention systolic blood pressure (SBP) in experimental and control groups. ANOVA revealed significant sex differences, with higher SBP in males than females (F(1,127) = 24.393, *p* < 0.001, η^2^_p_^G^ = 0.122, d ≈ 0.75). No main effects were found for intervention variant (F(4,127) = 1.697, *p* = 0.155, η^2^_p_^G^ = 0.037, d ≈ 0.39) or time (F(1,127) = 0.296, *p* = 0.587, η^2^_p_^G^ = 0.001, d ≈ 0.05).

Interaction terms were also non-significant, confirming baseline physiological states:

variant × time: F(4,127) = 0.254, *p* = 0.907, η^2^_p_^G^ = 0.002, d ≈ 0.09

sex × time: F(1,127) = 0.630, *p* = 0.429, η^2^_p_^G^ = 0.001, d ≈ 0.06

variant × sex × time: F(4,127) = 1.274, *p* = 0.284, η^2^_p_^G^ = 0.011, d ≈ 0.21

These results indicate that SBP changes over time were not significantly affected by HIIT duration or sex.

Post hoc tests (Bonferroni corrected) revealed no significant pre–post differences in systolic blood pressure in any group (all *p* = 1.000). Delta analyses also showed no significant effects for intervention variant (F = 0.25, *p* = 0.906), sex (F = 0.63, *p* = 0.429), or their interaction (F = 1.27, *p* = 0.283).

### 3.4. Diastolic Blood Pressure

**Figure 4:**
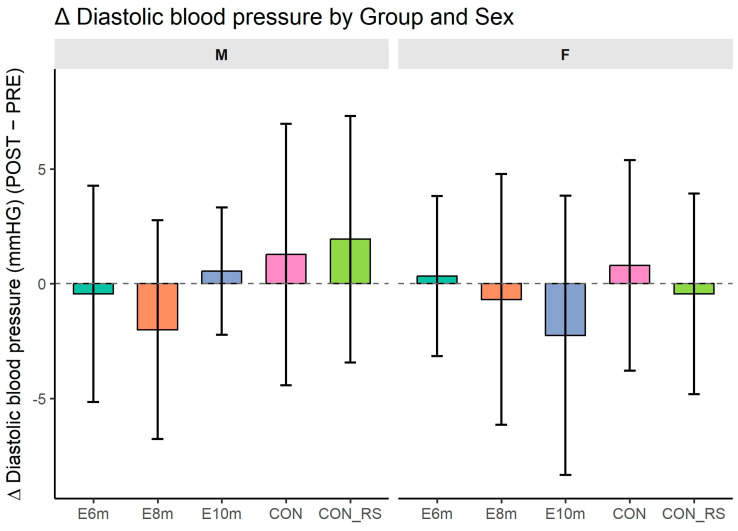
Changes (Δ = POST − PRE) in diastolic blood pressure (sbp) across intervention groups and sexes. Bars represent mean Δ values ± 95% CI; dashed line indicates zero change. Abbreviations: M—males; F—females; 6 min, 8 min, 10 min—experimental groups differing in session duration; CON—control group of non-responders; CON_Rs—control group of previous responders.

Figure 4 shows changes (Δ) between post- and pre-intervention diastolic blood pressure (DBP) in experimental and control groups. ANOVA revealed no main effect of time (F(1,127) = 0.016, *p* = 0.900, η^2^_p_^G^ < 0.001, d ≈ 0.01) and no effect of HIIT duration (F(4,127) = 0.391, *p* = 0.815, η^2^_p_^G^ = 0.009, d ≈ 0.19). The main effect of sex was marginally non-significant (F(1,127) = 3.717, *p* = 0.056, η^2^_p_^G^ = 0.022, d ≈ 0.30).

A significant sex × variant interaction was observed (F(4,127) = 3.551, *p* = 0.009, η^2^_p_^G^ = 0.078, d ≈ 0.58), indicating that training effects differed between males and females. Other interaction terms, including those related to time, were not statistically significant (all *p* > 0.05).

Post hoc tests showed no significant baseline differences in DBP (all *p* > 0.05). Although a decreasing trend was observed, no significant pre–post or post–follow-up changes were detected in either sex (all *p* = 1.000), and no differences were found compared with control groups (all *p* = 1.000). Likewise, there were no sex differences (all *p* = 1.000).

Delta analyses confirmed no significant effects of intervention variant (F = 0.39, *p* = 0.816), sex (F = 0.24, *p* = 0.627), or their interaction (F = 0.32, *p* = 0.866).

### 3.5. Cardiorespiratory Fitness (VO_2_max)

**Figure 5:**
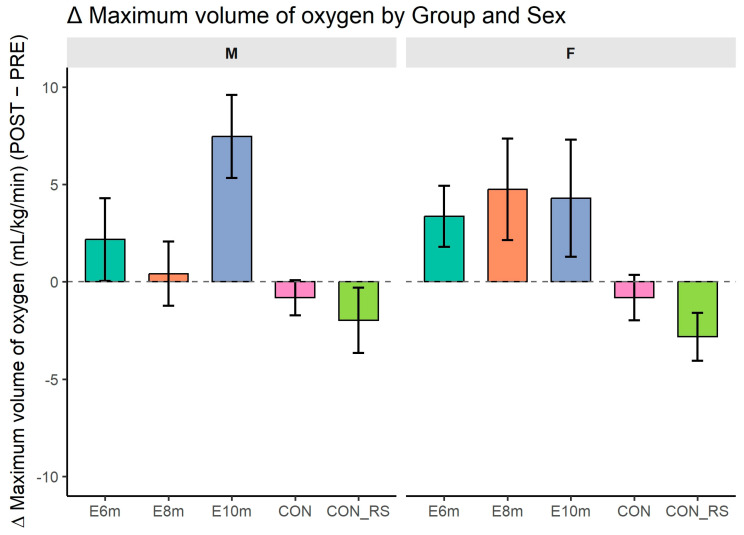
Changes (Δ = POST − PRE) in maximal oxygen consumption (VO_2_max) across intervention groups and sexes. Bars represent mean Δ values ± 95% CI; dashed line indicates zero change. Abbreviations: M—males; F—females; 6 min, 8 min, 10 min—experimental groups differing in session duration; CON—control group of non-responders; CON_Rs—control group of previous responders.

Figure 5 shows changes (Δ) between post- and pre-intervention cardiorespiratory fitness (VO_2_max) in experimental and control groups. ANOVA revealed a significant main effect of time (F(1,127) = 31.239, *p* < 0.001, η^2^_p_^G^ = 0.018, d ≈ 0.27) and sex (F(1,127) = 10.012, *p* = 0.002, η^2^_p_^G^ = 0.068, d ≈ 0.54). The effect of HIIT duration approached significance (F(4,127) = 2.401, *p* = 0.053, η^2^_p_^G^ = 0.065, d ≈ 0.52).

Significant interactions were observed for the following:

sex × duration variant (F(4,127) = 13.155, *p* < 0.001, η^2^_p_^G^ = 0.277, d ≈ 1.96),

duration × time (F(4,127) = 25.900, *p* < 0.001, η^2^_p_^G^ = 0.057, d ≈ 0.49),

sex × duration × time (F(4,127) = 4.496, *p* = 0.002, η^2^_p_^G^ = 0.010, d ≈ 0.20).

These results indicate that VO_2_max changes over time were influenced by both sex and session duration. No significant sex × time interaction was found (*p* > 0.05).

Post hoc comparisons (PRE vs. POST) revealed significant VO_2_max improvements in almost all groups. Among females, increases were observed in 10 min (*p* < 0.001), CON_RS (*p* < 0.001), 6 min (*p* = 0.004), 8 min (*p* = 0.030), and CON (*p* = 0.002) groups. Among males, improvements occurred in CON_RS (*p* < 0.001), 10 min (*p* < 0.001), 6 min (*p* = 0.007), 8 min (*p* = 0.030), and CON (*p* = 0.010). No group showed a decline in VO_2_max after intervention. The largest effects were seen in the 10 min HIIT and CON_RS groups.

Analysis of ΔVO_2_max showed significant effects of sex (F(1,127) = 5.174, *p* = 0.025) and HIIT duration (F(4,127) = 4.499, *p* = 0.002), as well as a sex × duration interaction (F(4,127) = 2.980, *p* = 0.022). These results indicate that VO_2_max improvements varied by both sex and training protocol.

Post hoc tests revealed no significant baseline differences between groups (all *p* > 0.05). Pre–post comparisons showed significant improvements in the 10 min HIIT and CON_RS groups among females (both *p* < 0.001), and in the CON_RS group among males (*p* = 0.002). Other pre–post comparisons were not significant (*p* > 0.05). Post-intervention values did not differ between experimental and control groups (*p* = 1.000), confirming minimal between-group differences.

Overall, while sex-specific effects were observed, only the sex × duration interaction reached statistical significance in the full ANOVA model. These findings suggest selective responsiveness to HIIT duration, with the strongest effects in females completing the 10 min or CON_RS protocols.

### 3.6. Classification for Rs-NRs

**Figure 6:**
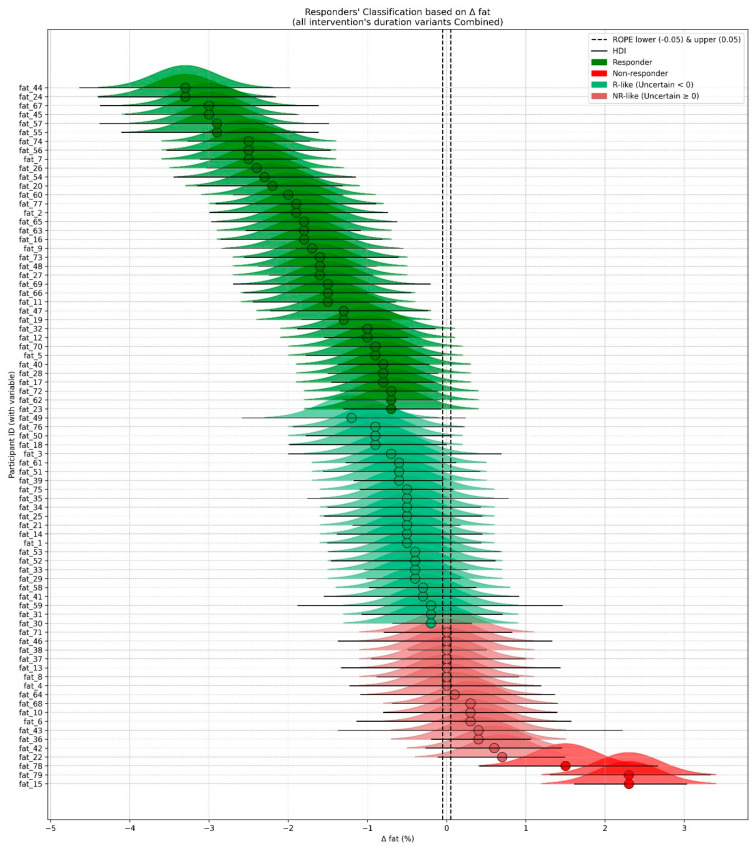
Individual Bayesian classification of training responses based on changes in body fat percentage (ΔBF%). Each participant is categorized as a responder (green), non-responder (red), or uncertain (shaded gradient) according to the 89% Highest Density Interval (HDI) and the Region of Practical Equivalence (ROPE, ±0.05). Abbreviations: HDI—Highest Density Interval; ROPE—Region of Practical Equivalence; Δ—change; BF%—body fat percentage; R—responder; NR—non-responder.

A Bayesian approach was used to classify participants as responders (Rs), non-responders (NRs), or uncertain cases, based on the Region of Practical Equivalence (ROPE) and the 89% Highest Density Interval (HDI). Figure 6 illustrates this classification for Δ BF%. The same procedure was applied to SBP, DBP, and VO_2_max, although detailed plots are not presented here, as the classification primarily served as the basis for subsequent regression and clustering analyses.

Table 3 shows the distribution of participants across response categories (Δ BF%, Δ SBP, Δ DBP, Δ VO_2_max) using a three-step Bayesian classification. Initially, participants were categorized as responders, non-responders, or uncertain based on whether their 89% HDI lay fully outside or overlapped the ROPE (±0.05). Uncertain cases were then reassigned as responder-like (R-like) or non-responder-like (NR-like) depending on the direction of change.

The final binary classification grouped responders and R-like cases as Bayesian responders (RsB) and non-responders with NR-like cases as Bayesian non-responders (NRsB). Across outcomes, RsB proportions ranged from 53.2% (VO_2_max) to 68.4% (DBP), while NRsB proportions ranged from 31.6% (DBP) to 46.8% (VO_2_max). A considerable proportion of uncertain cases (≈31–42%) highlighted the need for direction-based reassignment to strengthen binary modeling and subsequent analyses.

Table 4 summarizes improvements in participants who were previously non-responsive in one to four outcomes (BF%, SBP, DBP, VO_2_max). Among those non-responsive in a single outcome, 70.5% improved in that variable after the modified intervention. For participants non-responsive in two outcomes, 88.5% improved in at least one, though only 26.9% improved in both. In the group non-responsive in three outcomes, all participants (100%) improved in at least one, but only 14.3% improved in all. Of the two participants non-responsive in all four outcomes, both improved in at least one, and one improved across all variables.

These results indicate that many previously non-responsive individuals benefited from the modified intervention, although complete recovery across multiple outcomes was less common as the number of non-responsive domains increased.

Table 5 presents McNemar test results for participants initially classified as non-responders. A significant increase in responders was observed for SBP (*p* < 0.001) and VO_2_max (*p* < 0001 showing a significant change after the modified intervention) in these outcomes. No significant changes were found for BF% (*p* = 0.32) or DBP (*p* = 0.07), suggesting limited effects on these measures among non-responders.

Table 6 presents logistic regression results predicting improvement after the modified intervention. The number of previously non-responsive outcomes was a significant predictor (OR = 4.57, 95% CI: 1.23–16.94, *p* = 0.023). Each additional non-responsive variable was associated with a more than fourfold increase in the odds of becoming responsive. Neither sex nor intervention duration (8 or 10 min vs. 6 min) significantly predicted improvement. A non-significant trend toward higher odds of improvement was observed in females compared with males (OR = 0.30, 95% CI: 0.08–1.07, *p* = 0.064).

Table 7 presents results of the GEE analysis for predictors of improvement in previously non-responsive outcomes. The number of prior non-responsive variables was the only significant predictor (OR = 2.46, 95%, CI: 1.26–4.76, *p* = 0.008), showing that each additional non-response more than doubled the likelihood of later improvement. Neither sex (OR = 0.52, *p* = 0.104) nor intervention duration (8 min: OR = 0.94, *p* = 0.924; 10 min: OR = 0.61, *p* = 0.318) had significant effects. These findings confirm that prior non-responsiveness strongly predicts future improvement, independent of sex or training duration.

## 4. Discussion

This study examined the effects of three HIIT session durations (6, 8, and 10 min) on BF%, blood pressure, and VO_2_max in adolescents. All interventions provided positive changes, with the 10 min protocol yielding the most consistent VO_2_max improvements, suggesting a dose–response effect in aerobic capacity. No significant differences between durations were observed for BF% or blood pressure. Using a Bayesian framework, we found substantial inter-individual variability in responsiveness, with 53–68% of participants classified as responders depending on the outcome. Importantly, many adolescents previously identified as non-responders improved after the modified intervention, particularly in VO_2_max and SBP, where more than 70% showed positive changes. These findings highlight the value of individualized monitoring and suggest that non-response can often be overcome when training parameters are adjusted. The present findings extend prior evidence by demonstrating that individualized dose modification can elicit positive adaptations even in participants who previously failed to respond to standard HIIT protocols. The findings of the present study extend the existing evidence on the health benefits of HIIT in adolescents by introducing a dose–response perspective within the school environment. Unlike previous research typically employing standardized or longer HIIT sessions, our intervention compared micro-duration (6, 8, and 10 min) Tabata-type protocols implemented during routine physical education lessons. Importantly, the study incorporated daily step monitoring and responder/non-responder analysis, providing a more comprehensive understanding of inter-individual variability in adaptations related to blood pressure and VO_2_max improvements.

Minor improvements in VO_2_max were also noted in control groups, likely reflecting regular physical education activity and test familiarization effects rather than a training-specific adaptation. In the experimental groups, repeated high-intensity efforts likely enhanced cardiovascular efficiency (increased stroke volume and oxygen delivery) and mitochondrial oxidative capacity, explaining the greater VO_2_max gains. In contrast, the minor improvements in the control group may also reflect natural maturation processes typical for mid-adolescence, including increases in cardiac output and hemoglobin concentration.

It should be noted that while group-level analyses did not reveal significant changes in blood pressure, individual Bayesian classification identified several previously non-responsive participants who showed meaningful reductions after the modified intervention. The consistent improvements in VO_2_max observed with the 10 min sessions may suggest that a higher accumulated training volume and metabolic load could be important to surpass the physiological threshold required for cardiopulmonary adaptation. Although this pattern aligns with a potential dose–response trend, the lack of significant between-group differences warrants cautious interpretation. [9,10]. This interpretation is supported by systematic reviews showing that longer or higher-volume HIIT protocols lead to greater aerobic fitness improvements in adolescents [20,21]. Such effects may depend on individual characteristics, as inter-individual variability in adaptation is influenced by differences in mitochondrial efficiency, autonomic regulation, and genetic predisposition [20]. Accordingly, the improvement observed in some non-responders after the longer sessions indicates that insufficient training stimulus could partly explain the initial lack of adaptation [20,22,23]. Together, these findings highlight the dose–response nature of HIIT and emphasize the importance of individualized adjustments to overcome early non-responsiveness.

Compared with other school-based HIIT studies [4,5,10,24], our 10 min sessions produced VO_2_max gains similar to or greater than those from longer interventions, showing that relatively short but intense protocols can be effective [20]. In contrast, we found no significant between-group differences in BF% or blood pressure, unlike some studies on obese or low-active individuals. This may relate to initial fitness levels, adherence, or intervention length [25]. Motivational aspects such as enjoyment and peer interaction, reported as important mediators of adolescent HIIT [26,27], may also have supported engagement in our school-based context, helping some non-responders to improve. Overall, adolescent responsiveness appears to vary substantially with fitness level and intervention characteristics.

Several limitations should be noted. Reliance on group-derived data may not capture individual physiological adaptations, such as hormonal responses. The study did not stratify participants by maturation stage or BMI, which are important confounders influencing training responsiveness in adolescents. Although the follow-up intervention was conducted six months after the initial trial using the same participants, minor maturational changes may have occurred during this period and could partly contribute to variability in responsiveness. However, all participants remained within the same school grade and age cohort, which minimized the impact of growth-related differences. This may partly explain the observed inter-individual variability. Additionally, the absence of a long-term follow-up limits the understanding of whether the improvements were sustained over time. Future research should integrate molecular biomarkers and individualized dose quantification (e.g., TRIMP) to better explain the mechanisms of responsiveness in adolescents.

## 5. Conclusions

The findings demonstrate that even very short, school-based HIIT protocols can elicit meaningful improvements in key health indicators among adolescents. Longer sessions tended to produce more consistent aerobic adaptations, while considerable inter-individual variability indicated that non-response is often transient and can be modified through appropriate training adjustments. These results underscore the value of individualized HIIT prescription and systematic monitoring to optimize adaptations and promote sustained engagement in physical activity within the limited time available during physical education classes.

## Figures and Tables

**Table 1:** Descriptive statistics of the baseline anthropometric measurements in males and females from experimental and control groups.

		Males				Females		
Variable	Mean	.-95%CI	95%CI	SD	Mean	.-95%CI	95%CI	SD
	E6m	n = 16				E6m	n = 12	
Age [y]	16.52	16.25	16.78	0.49	16.50	16.19	16.82	0.50
Body height [cm]	177.56	175.06	180.07	4.70	163.48	160.62	166.33	4.49
Body weight [kg]	66.03	59.80	72.26	11.69	51.53	48.40	54.65	4.92
Body mass index [kg/m^2^]	20.95	19.00	22.90	3.66	19.34	17.84	20.85	2.37
	E8m	n = 12				E8m	n = 16	
Age [y]	16.54	16.21	16.86	0.51	16.82	16.59	17.06	0.45
Body height [cm]	177.12	174.95	179.28	3.41	165.25	162.10	168.40	5.92
Body weight [kg]	69.77	63.13	76.41	10.45	57.43	52.90	61.95	8.49
Body mass index [kg/m^2^]	22.25	20.10	24.41	3.39	21.06	19.35	22.77	3.21
	E10m	n = 11				E10m	n = 12	
Age [y]	16.77	16.47	17.07	0.44	16.59	16.25	16.92	0.52
Body height [cm]	178.19	172.76	183.62	8.08	162.47	159.54	165.39	4.61
Body weight [kg]	67.80	60.15	75.45	11.39	52.74	48.30	57.18	6.99
Body mass index [kg/m^2^]	21.25	19.49	23.02	2.62	20.02	18.24	21.81	2.80
	CON	n = 11				CON	n = 15	
Age [y]	16.68	16.27	17.10	0.62	16.99	16.66	17.31	0.59
Body height [cm]	176.91	172.12	181.70	7.13	161.80	148.28	175.32	24.42
Body weight [kg]	63.85	53.92	73.79	14.78	57.28	52.94	61.62	7.84
Body mass index [kg/m^2^]	20.24	17.78	22.71	3.67	25.60	14.13	37.07	20.71
	CON_Rs	n = 16				CON_Rs	n = 16	
Age [y]	16.91	16.64	17.18	0.50	16.65	16.49	16.80	0.29
Body height [cm]	179.99	176.75	183.22	6.08	166.54	163.42	169.67	5.86
Body weight [kg]	70.31	66.74	73.89	6.71	62.67	56.47	68.87	11.63
Body mass index [kg/m^2^]	21.77	20.46	23.08	2.46	22.68	20.22	25.14	4.62

Footnote: Two-way ANOVA confirmed significant sex differences in body height and body weight (*p* < 0.01, η^2^_p_^G^ values shown in text), but no significant group or interaction effects (all *p* > 0.05). Values are presented as mean ± SD.

**Table 2:** Descriptive statistics of the baseline, post-intervention, and differences (∆) in BF%, systolic and diastolic blood pressure, and VO_2_max.

			Males				Females		
Variable		Mean	-95%CI	95%CI	SD	Mean	-95%CI	95%CI	SD
		E6m	n = 16				E6m	n = 12	
	PRE	17.01	14.51	19.50	4.69	24.23	21.90	26.57	3.67
Body fat [%]	POST	16.16	13.47	18.85	5.05	23.33	20.80	25.85	3.98
	Δ	−0.84	−1.50	−0.19	1.23	−0.91	−1.57	−0.25	1.04
	PRE	119.56	116.19	122.94	6.33	107.58	101.85	113.32	9.03
Systolic blood pressure [mmHG]	POST	117.19	113.24	121.13	7.40	108.25	103.07	113.43	8.15
	Δ	−2.38	−5.77	1.02	6.37	0.67	−6.34	7.68	11.03
	PRE	71.75	67.64	75.86	7.72	75.25	70.81	79.69	7.00
Diastolic blood pressure [mmHG]	POST	71.31	66.21	76.41	9.57	75.58	70.24	80.93	8.41
	Δ	−0.44	−5.15	4.27	8.84	0.33	−3.15	3.82	5.48
	PRE	39.65	36.39	42.92	6.13	29.60	26.90	32.30	4.25
VO_2_max [/kg/min]	POST	41.83	38.97	44.68	5.36	32.96	30.15	35.77	4.43
	Δ	2.17	0.05	4.30	3.99	3.36	1.79	4.93	2.47
		E8m	n = 12				E8m	n = 16	
	PRE	16.48	13.36	19.61	4.92	25.58	22.59	28.58	5.62
Body fat [%]	POST	16.24	13.07	19.42	5.00	24.27	21.09	27.45	5.97
	Δ	−0.24	−0.53	0.05	0.46	−1.31	−1.93	−0.70	1.16
	PRE	122.67	117.79	127.55	7.68	108.06	101.32	114.80	12.65
Systolic blood pressure [mmHG]	POST	121.00	112.22	129.78	13.82	105.06	90.14	119.99	28.01
	Δ	−1.67	−8.01	4.68	9.98	−3.00	−15.90	9.90	24.20
	PRE	77.67	72.55	82.79	8.06	74.56	71.66	77.46	5.44
Diastolic blood pressure [mmHG]	POST	75.67	70.93	80.40	7.45	73.88	68.35	79.40	10.37
	Δ	−2.00	−6.76	2.76	7.50	−0.69	−6.15	4.77	10.24
	PRE	27.65	26.04	29.25	2.53	33.40	30.50	36.30	5.44
VO_2_max [mL/kg/min]	POST	28.06	25.50	30.62	4.03	38.15	35.22	41.08	5.51
	Δ	0.41	−1.24	2.07	2.60	4.75	2.14	7.35	4.89
		E10m	n = 11				E10m	n = 12	
	PRE	17.55	14.51	20.60	4.54	25.19	21.05	29.34	6.52
Body fat [%]	POST	16.62	13.54	19.69	4.58	24.29	20.17	28.41	6.49
	Δ	−0.94	−1.50	−0.37	0.85	−0.90	−1.94	0.14	1.63
	PRE	116.27	106.98	125.57	13.84	113.33	103.82	122.84	14.97
Systolic blood pressure [mmHG]	POST	117.73	111.16	124.30	9.78	114.08	106.23	121.94	12.36
	Δ	1.45	−6.55	9.46	11.92	0.75	−6.52	8.02	11.43
	PRE	70.09	66.16	74.02	5.86	82.00	74.60	89.40	11.65
Diastolic blood pressure [mmHG]	POST	70.64	65.45	75.83	7.72	79.75	72.32	87.18	11.69
	Δ	0.55	−2.23	3.32	4.13	−2.25	−8.33	3.83	9.56
	PRE	27.70	25.22	30.19	3.70	32.43	29.22	35.63	5.05
VO_2_max [mL/kg/min]	POST	35.16	33.28	37.04	2.80	36.71	31.45	41.97	8.27
	Δ	7.46	5.33	9.59	3.17	4.28	1.28	7.29	4.73
		CON	n = 11				CON	n = 15	
	PRE	15.38	11.27	19.49	6.11	27.00	23.42	30.58	6.47
Body fat [%]	POST	14.63	11.19	18.07	5.12	27.07	23.69	30.45	6.11
	Δ	−0.75	−3.82	2.31	4.56	0.07	−0.64	0.78	1.28
	PRE	111.82	100.13	123.50	17.39	110.13	105.19	115.07	8.92
Systolic blood pressure [mmHG]	POST	111.82	104.27	119.37	11.24	107.27	102.38	112.15	8.82
	Δ	0.00	−8.50	8.50	12.66	−2.87	−6.66	0.92	6.84
	PRE	71.73	63.29	80.16	12.55	75.33	69.77	80.89	10.04
Diastolic blood pressure [mmHG]	POST	73.00	68.70	77.30	6.40	76.13	72.71	79.55	6.17
	Δ	1.27	−4.42	6.97	8.47	0.80	−3.79	5.39	8.28
	PRE	40.07	36.07	44.07	5.95	30.08	27.34	32.82	4.95
VO_2_max [mL/kg/min]	POST	39.25	34.99	43.51	6.35	29.27	26.33	32.21	5.31
	Δ	−0.82	−1.72	0.08	1.33	−0.81	−1.97	0.35	2.09
		CON_Rs	n = 16				CON_Rs	n = 16	
	PRE	16.73	14.29	19.17	4.57	29.48	25.00	33.95	8.39
Body fat [%]	POST	16.74	14.54	18.94	4.13	29.02	25.20	32.84	7.17
	Δ	0.01	−0.60	0.62	1.14	−0.46	−2.52	1.61	3.87
	PRE	128.06	121.02	135.11	13.22	107.00	99.58	114.42	13.93
Systolic blood pressure [mmHG]	POST	122.69	116.95	128.42	10.76	112.94	105.75	120.12	13.49
	Δ	−5.38	−12.23	1.48	12.86	5.94	−3.41	15.29	17.55
	PRE	75.25	70.37	80.13	9.16	73.50	70.22	76.78	6.16
Diastolic blood pressure [mmHG]	POST	77.19	71.89	82.48	9.94	73.06	70.34	75.79	5.12
	Δ	1.94	−3.43	7.30	10.07	−0.44	−4.81	3.93	8.20
	PRE	40.14	35.30	44.97	9.07	32.97	28.81	37.12	7.80
VO_2_max [mL/kg/min]	POST	38.15	33.49	42.81	8.75	30.15	26.39	33.91	7.06
	Δ	−1.99	−3.66	−0.31	3.15	−2.82	−4.05	−1.59	2.31

Footnote: Statistical comparisons were performed using mixed-effects ANOVA (sex × group × time). Significant results and effect sizes (η^2^_p_^G^, Cohen’s d) are presented in the text (Section 3). Values are shown as mean ± SD. PRE—pre-intervention, POST—post-intervention, Δ (delta)—change between post-intervention and pre-intervention, VO_2_max—maximal oxygen consumption/uptake.

**Table 3:** Distribution of participants across Bayesian response classifications for each outcome variable. Initial classification (responder, non-responder, uncertain) was followed by directional reallocation of uncertain cases into responder-like (R-like) or non-responder-like (NR-like) groups. The final binary classification (RsB vs. NRsB) was used in regression analyses.

	Responder	Non-Responder	Uncertain	→ R-Like	→ NR-Like	Final RsB	Final NRsB
Body fat percentage	30 (38.0%)	16 (20.3%)	33 (41.8%)	20 (25.3%)	13 (16.5%)	50 (63.3%)	29 (36.7%)
Systolic blood pressure	37 (46.8%)	17 (21.5%)	25 (31.6%)	14 (17.7%)	11 (13.9%)	51 (64.6%)	28 (35.4%)
Diastolic blood pressure	37 (46.8%)	15 (19.0%)	27 (34.2%)	17 (21.5%)	10 (12.7%)	54 (68.4%)	25 (31.6%)
VO_2_max	26 (32.9%)	24 (30.4%)	29 (36.7%)	16 (20.3%)	13 (16.5%)	42 (53.2%)	37 (46.8%)

**Table 4:** Improvement in same non-responsive variables.

NRs (Previous Project)	Total	Improved in Any N (%)	Improved in All
Body fat percentage	44	31 (70.5%)	31 (70.5%)
Systolic blood pressure	26	23 (88.5%)	7 (26.9%)
Diastolic blood pressure	7	7 (100.0%)	1 (14.3%)
VO_2_max	2	2 (100.0%)	1 (50.0%)

**Table 5:** Results of McNemar’s test comparing changes in individual responsiveness status between the initial and modified interventions across outcome variables (BF%, SBP, DBP, VO_2_).

Variable	Non-Response → Response	Response → Non-Response	McNemar’s *χ*^2^	*p*-Value	Statistic
Body fat percentage	22	15	0.97	0.324	15
Systolic blood pressure	8	33	14.05	0.000	8
Diastolic blood pressure	10	21	3.23	0.071	10
VO_2_max	40	6	23.67	0.000	6

**Table 6:** Results of logistic regression predicting the probability of improvement in non-responsive outcomes based on sex, intervention duration, and the number of non-responsive variables in the initial experiment.

	b	Error	z	*p* > |z|	OR	−95%CI	+95%CI
Intercept	0.13	1.09	0.12	0.908	1.14	0.13	9.58
Sex [M]	−1.2	0.65	−1.85	0.064	0.30	0.08	1.07
E8m	−0.07	0.75	−0.09	0.929	0.93	0.21	4.10
E10m	−0.48	0.79	−0.61	0.539	0.62	0.13	2.89
Non-responsive outcome	1.52	0.67	2.27	0.023	4.57	1.23	16.95

Footnote: OR—Odds Ratio; M—comparison between males (M) and females (reference); E8m—comparison between 8 min intervention and 6 min intervention (reference); E10m—comparison between 10 min intervention and 6 min intervention (reference).

**Table 7:** Results of the Generalized Estimating Equation (GEE) model predicting the likelihood of improvement in previously non-responsive outcomes, including Odds Ratios (ORs), 95% confidence intervals, and *p*-values.

	Coef	Std, Err	z	*p*_Value	OR	CI_Lower	CI_Upper
Intercept	0.70	0.49	1.43	0.153	2.01	0.77	5.26
Sex [M]	−0.66	0.31	−2.13	0.033	0.52	0.28	0.95
E8m	−0.06	0.34	−0.18	0.860	0.94	0.48	1.83
E10m	−0.50	0.37	−1.35	0.177	0.61	0.29	1.25
Non-responsive outcome	0.90	0.30	3.00	0.003	2.46	1.37	4.43

Footnote: OR—Odds Ratio; M—comparison between males (M) and females (reference); 8—comparison between 8 min intervention and 6 min intervention (reference); 10—comparison between 10 min intervention and 6 min intervention (reference).

## Data Availability

The raw data supporting the conclusions of this article will be made available by the authors on request.
